# Tetra­methyl 5,5′-[4,5-dicyano-1,2-phenyl­enebis(­oxy)]diisophthalate chloro­form monosolvate

**DOI:** 10.1107/S1600536811024718

**Published:** 2011-06-30

**Authors:** Fei Yang, Fanjun Meng, Xiaomei Zhang, Ming Bai

**Affiliations:** a Marine College, Shandong University at Weihai, Weihai 264209, People’s Republic of China; b School of Chemistry & Chemical Technology, Shandong University, Jinan 250100, People’s Republic of China

## Abstract

In the title compound, C_28_H_20_N_2_O_10_·CHCl_3_, the phen­oxy rings are inclined to the central phenyl ring at dihedral angles of 84.71 (13) and 80.56 (13)°. In the crystal, pairs of weak inter­molecular C—H⋯O hydrogen bonds link mol­ecules related by an inversion center, forming dimers. There are also C—H⋯π inter­actions present.

## Related literature

For general structural and background information on phthalocyanines, including properties and appplications, see: Kobayashi (2001 ▶); LukCentyanets (1999 ▶); Suda *et al.* (2009 ▶); Zhang *et al.* (2009 ▶). For the synthesis of the title compound, see: del Rey *et al.* (1998 ▶). For the crystal structure of a similar compound, dimethyl 2,2′-(4,5-dicyano-*o*-phenyl­enedi­oxy)dibenzoate, see: Ocak *et al.* (2004 ▶).
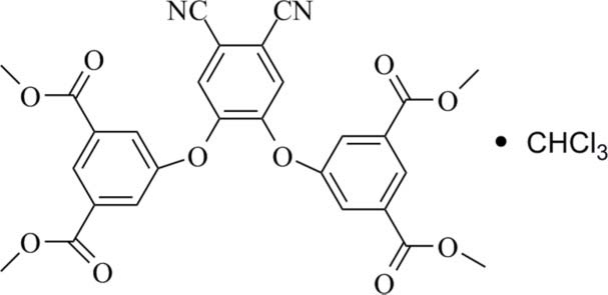


## Experimental

### 

#### Crystal data

C_28_H_20_N_2_O_10_·CHCl_3_
*M* *_r_* = 663.85
Triclinic, 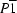

*a* = 9.9223 (13) Å
*b* = 11.4374 (15) Å
*c* = 13.9398 (19) Å
α = 96.860 (2)°
β = 94.578 (2)°
γ = 105.326 (2)°
*V* = 1504.5 (3) Å^3^
*Z* = 2
Mo *K*α radiation
μ = 0.37 mm^−1^
*T* = 298 K
0.15 × 0.12 × 0.05 mm

#### Data collection

Bruker SMART CCD area-detector diffractometer
7568 measured reflections
5257 independent reflections
3557 reflections with *I* > 2σ(*I*)
*R*_int_ = 0.029

#### Refinement

*R*[*F* ^2^ > 2σ(*F* ^2^)] = 0.056
*wR*(*F* ^2^) = 0.159
*S* = 1.08
5257 reflections
397 parameters
H-atom parameters constrained
Δρ_max_ = 0.46 e Å^−3^
Δρ_min_ = −0.49 e Å^−3^

### 

Data collection: *SMART* (Bruker, 2007 ▶); cell refinement: *SAINT-Plus* (Bruker, 2007 ▶); data reduction: *SAINT-Plus*; program(s) used to solve structure: *SHELXS97* (Sheldrick, 2008 ▶); program(s) used to refine structure: *SHELXL97* (Sheldrick, 2008 ▶); molecular graphics: *SHELXTL* (Sheldrick, 2008 ▶); software used to prepare material for publication: *SHELXTL*.

## Supplementary Material

Crystal structure: contains datablock(s) global, I. DOI: 10.1107/S1600536811024718/su2272sup1.cif

Structure factors: contains datablock(s) I. DOI: 10.1107/S1600536811024718/su2272Isup2.hkl

Supplementary material file. DOI: 10.1107/S1600536811024718/su2272Isup3.cml

Additional supplementary materials:  crystallographic information; 3D view; checkCIF report

## Figures and Tables

**Table 1:** Hydrogen-bond geometry (Å, °) *Cg*2 is the centroid of the C11–C16 ring.

*D*—H⋯*A*	*D*—H	H⋯*A*	*D*⋯*A*	*D*—H⋯*A*
C15—H15⋯O7^i^	0.93	2.60	3.455 (4)	154
C29—H29⋯O3^ii^	0.98	2.26	3.182 (5)	157
C19—H19*A*⋯*Cg*2^iii^	0.98	2.90	3.709 (4)	143

Symmetry codes: (i) 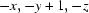; (ii) 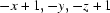; (iii) 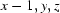.

**Fig. 1.**
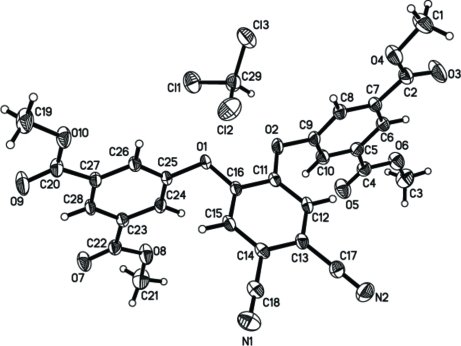
A view of the molecular structure of the title compound with the atom labelling scheme. Displacement ellipsoids are drawn at the 30% probability level.

## supplementary crystallographic information

### Comment

Dicyano compounds have been widely used to synthesize many useful materials such as phthalocyanines. Phthalocyanines are an interesting class of compounds, with increasingly diverse industrial and biomedical applications, for instance as dyes and pigments, materials for optical storage (Kobayashi *et al.* 2001), photodynamic therapy agents (LukCentyanets *et al.* 1999), catalysis (Suda *et al.* 2009), and corrosion inhibitors (Zhang *et al.* 2009). The title compound was prepared according to the method reported in the literature (del Rey *et al.*, 1998), and its crystal structure is described herein. The crystal structure of a similar compound, Dimethyl 2,2'-(4,5-dicyano-o-phenylenedioxy)dibenzoate, has been described by (Ocak *et al.*, 2004).

The molecular structure of the title compound is shown in Fig. 1. It has a kite-like configuration with the aromatic rings and two cyanide groups being the head and the substituted 3,5-Bismethoxycarbonyl phenoxy groups being the tails. The mean planes of the phenoxy rings [A = (C5-C10) and C = (C23-C28)] are inclinded to the mean plane of the central phenyl ring [B = (C11-C16)], with dihedral angles of A/B = 84.71 (13) ° and C/B = 80.56 (13) °, while planes A and C are inclined to one another by 11.93 (13) °.

In the crystal weak intermolecular C—H···O hydrogen bonds link molecules related by an inversion center to form dimers (Table 1). There is also a C-H···π interaction present in the crystal structure (Table 1).

### Experimental

The title compound was prepared according to a published procedure (del Rey *et al.*, 1998). Colourless block-like crystals, suitable for X-ray diffraction analysis, were obtained by slow evaporation of a solution of the title compound in chloroform.

### Refinement

H-atoms were placed in calculated positions and refined using a riding-model approximation: C—H = 0.93 and 0.97 Å, for CH(aromatic) and CH_3_ H-atoms, respectively, with *U*_iso_ = k × *U*_eq_(C) where k = 1.5 for CH_3_ H-atoms and k = 1.2 for CH(aromatic) H-atoms.

### Crystal data

**Table:**

C_28_H_20_N_2_O_10_·CHCl_3_	*Z* = 2
*M**_r_* = 663.85	*F*(000) = 680
Triclinic, *P*1	*D*_x_ = 1.465 Mg m^−^^3^
Hall symbol: -P 1	Mo *K*α radiation, λ = 0.71073 Å
*a* = 9.9223 (13) Å	Cell parameters from 1932 reflections
*b* = 11.4374 (15) Å	θ = 2.7–23.6°
*c* = 13.9398 (19) Å	µ = 0.37 mm^−^^1^
α = 96.860 (2)°	*T* = 298 K
β = 94.578 (2)°	Block, colourless
γ = 105.326 (2)°	0.15 × 0.12 × 0.05 mm
*V* = 1504.5 (3) Å^3^	

### Data collection

**Table:**

Bruker SMART CCD area-detector diffractometer	3557 reflections with *I* > 2σ(*I*)
Radiation source: fine-focus sealed tube	*R*_int_ = 0.029
graphite	θ_max_ = 25.0°, θ_min_ = 1.5°
φ and ω scans	*h* = −11→11
7568 measured reflections	*k* = −7→13
5257 independent reflections	*l* = −16→16

### Refinement

**Table:**

Refinement on *F*^2^	Primary atom site location: structure-invariant direct methods
Least-squares matrix: full	Secondary atom site location: difference Fourier map
*R*[*F*^2^ > 2σ(*F*^2^)] = 0.056	Hydrogen site location: inferred from neighbouring sites
*wR*(*F*^2^) = 0.159	H-atom parameters constrained
*S* = 1.08	*w* = 1/[σ^2^(*F*_o_^2^) + (0.0657*P*)^2^ + 0.6098*P*] where *P* = (*F*_o_^2^ + 2*F*_c_^2^)/3
5257 reflections	(Δ/σ)_max_ < 0.001
397 parameters	Δρ_max_ = 0.46 e Å^−^^3^
0 restraints	Δρ_min_ = −0.49 e Å^−^^3^

### Special details

**Table:**

Geometry. All e.s.d.'s (except the e.s.d. in the dihedral angle between two l.s. planes) are estimated using the full covariance matrix. The cell e.s.d.'s are taken into account individually in the estimation of e.s.d.'s in distances, angles and torsion angles; correlations between e.s.d.'s in cell parameters are only used when they are defined by crystal symmetry. An approximate (isotropic) treatment of cell e.s.d.'s is used for estimating e.s.d.'s involving l.s. planes.
Refinement. Refinement of *F*^2^ against ALL reflections. The weighted *R*-factor *wR* and goodness of fit *S* are based on *F*^2^, conventional *R*-factors *R* are based on *F*, with *F* set to zero for negative *F*^2^. The threshold expression of *F*^2^ > σ(*F*^2^) is used only for calculating *R*-factors(gt) *etc*. and is not relevant to the choice of reflections for refinement. *R*-factors based on *F*^2^ are statistically about twice as large as those based on *F*, and *R*- factors based on ALL data will be even larger.

### Fractional atomic coordinates and isotropic or equivalent isotropic displacement parameters (Å^2^)

**Table:**

	*x*	*y*	*z*	*U*_iso_*/*U*_eq_	
Cl1	0.33835 (13)	0.52563 (10)	0.60702 (9)	0.0890 (4)	
Cl2	0.27318 (15)	0.33393 (13)	0.44608 (10)	0.1086 (5)	
Cl3	0.25891 (16)	0.27612 (12)	0.63996 (12)	0.1147 (5)	
O1	0.0551 (2)	0.25073 (18)	0.19677 (15)	0.0442 (5)	
O2	0.2198 (2)	0.12734 (18)	0.26465 (14)	0.0449 (5)	
O3	0.3694 (3)	−0.2971 (3)	0.4139 (3)	0.0968 (11)	
O4	0.1564 (2)	−0.2758 (2)	0.37967 (19)	0.0617 (6)	
O5	0.7479 (3)	0.2270 (2)	0.3509 (2)	0.0726 (7)	
O6	0.7752 (2)	0.0552 (2)	0.39934 (19)	0.0641 (7)	
O7	0.2002 (2)	0.7618 (2)	0.09592 (19)	0.0631 (7)	
O8	0.3489 (2)	0.6556 (2)	0.1366 (2)	0.0668 (7)	
O9	−0.3096 (2)	0.5709 (2)	0.0965 (2)	0.0718 (8)	
O10	−0.3797 (2)	0.3866 (2)	0.14128 (19)	0.0609 (7)	
N1	0.0918 (4)	0.1109 (3)	−0.2174 (2)	0.0726 (9)	
N2	0.3628 (4)	−0.0670 (3)	−0.1112 (2)	0.0697 (9)	
C1	0.0951 (4)	−0.3963 (3)	0.4050 (3)	0.0742 (11)	
H1A	−0.0057	−0.4147	0.3956	0.111*	
H1B	0.1255	−0.3975	0.4719	0.111*	
H1C	0.1247	−0.4564	0.3643	0.111*	
C2	0.2951 (4)	−0.2372 (3)	0.3889 (2)	0.0520 (8)	
C3	0.9268 (4)	0.1075 (4)	0.4124 (3)	0.0765 (12)	
H3A	0.9712	0.0493	0.4353	0.115*	
H3B	0.9512	0.1807	0.4591	0.115*	
H3C	0.9584	0.1269	0.3514	0.115*	
C4	0.6988 (3)	0.1245 (3)	0.3676 (2)	0.0480 (8)	
C5	0.5452 (3)	0.0611 (3)	0.3552 (2)	0.0423 (7)	
C6	0.4918 (3)	−0.0554 (3)	0.3781 (2)	0.0427 (7)	
H6	0.5526	−0.0971	0.4026	0.051*	
C7	0.3471 (3)	−0.1105 (3)	0.3645 (2)	0.0406 (7)	
C8	0.2562 (3)	−0.0503 (3)	0.3263 (2)	0.0414 (7)	
H8	0.1597	−0.0868	0.3163	0.050*	
C9	0.3125 (3)	0.0655 (3)	0.3032 (2)	0.0405 (7)	
C10	0.4542 (3)	0.1227 (3)	0.3169 (2)	0.0440 (7)	
H10	0.4889	0.2009	0.3009	0.053*	
C11	0.1995 (3)	0.1196 (2)	0.1661 (2)	0.0344 (6)	
C12	0.2607 (3)	0.0520 (2)	0.1030 (2)	0.0383 (7)	
H12	0.3180	0.0072	0.1272	0.046*	
C13	0.2363 (3)	0.0511 (2)	0.0030 (2)	0.0370 (7)	
C14	0.1470 (3)	0.1163 (2)	−0.0330 (2)	0.0388 (7)	
C15	0.0845 (3)	0.1825 (2)	0.0309 (2)	0.0390 (7)	
H15	0.0235	0.2243	0.0069	0.047*	
C16	0.1129 (3)	0.1864 (2)	0.1303 (2)	0.0334 (6)	
C17	0.3050 (3)	−0.0161 (3)	−0.0612 (2)	0.0461 (8)	
C18	0.1171 (3)	0.1129 (3)	−0.1363 (3)	0.0475 (8)	
C19	−0.5227 (3)	0.3945 (4)	0.1327 (4)	0.0805 (13)	
H19A	−0.5841	0.3203	0.1474	0.121*	
H19B	−0.5497	0.4057	0.0675	0.121*	
H19C	−0.5296	0.4627	0.1774	0.121*	
C20	−0.2831 (3)	0.4828 (3)	0.1232 (2)	0.0460 (8)	
C21	0.4657 (4)	0.7573 (3)	0.1234 (3)	0.0764 (12)	
H21A	0.5525	0.7368	0.1364	0.115*	
H21B	0.4659	0.8286	0.1674	0.115*	
H21C	0.4560	0.7738	0.0576	0.115*	
C22	0.2215 (3)	0.6710 (3)	0.1206 (2)	0.0444 (7)	
C23	0.1085 (3)	0.5622 (3)	0.1353 (2)	0.0383 (7)	
C24	0.1361 (3)	0.4546 (3)	0.1580 (2)	0.0423 (7)	
H24	0.2280	0.4489	0.1648	0.051*	
C25	0.0262 (3)	0.3570 (2)	0.1704 (2)	0.0369 (7)	
C26	−0.1108 (3)	0.3622 (2)	0.1620 (2)	0.0378 (7)	
H26	−0.1836	0.2959	0.1717	0.045*	
C27	−0.1380 (3)	0.4696 (2)	0.1384 (2)	0.0368 (6)	
C28	−0.0290 (3)	0.5684 (2)	0.1264 (2)	0.0388 (7)	
H28	−0.0484	0.6401	0.1121	0.047*	
C29	0.3476 (4)	0.3780 (3)	0.5670 (3)	0.0693 (10)	
H29	0.4465	0.3775	0.5714	0.083*	

### Atomic displacement parameters (Å^2^)

**Table:**

	*U*^11^	*U*^22^	*U*^33^	*U*^12^	*U*^13^	*U*^23^
Cl1	0.1033 (9)	0.0602 (6)	0.0974 (8)	0.0206 (6)	−0.0056 (7)	0.0035 (6)
Cl2	0.1101 (10)	0.1139 (10)	0.0909 (9)	0.0277 (8)	0.0109 (7)	−0.0198 (7)
Cl3	0.1220 (11)	0.0944 (9)	0.1517 (13)	0.0433 (8)	0.0374 (10)	0.0686 (9)
O1	0.0507 (12)	0.0411 (11)	0.0573 (13)	0.0295 (10)	0.0204 (10)	0.0252 (10)
O2	0.0547 (13)	0.0479 (12)	0.0438 (12)	0.0335 (10)	0.0024 (10)	0.0111 (9)
O3	0.0627 (17)	0.084 (2)	0.172 (3)	0.0375 (16)	0.0266 (19)	0.082 (2)
O4	0.0492 (14)	0.0522 (14)	0.0856 (17)	0.0126 (11)	−0.0005 (12)	0.0264 (12)
O5	0.0580 (16)	0.0530 (16)	0.105 (2)	0.0099 (12)	0.0025 (14)	0.0214 (15)
O6	0.0420 (13)	0.0696 (16)	0.0866 (18)	0.0209 (12)	0.0016 (12)	0.0266 (13)
O7	0.0543 (14)	0.0385 (13)	0.103 (2)	0.0147 (11)	0.0114 (13)	0.0288 (13)
O8	0.0338 (12)	0.0472 (13)	0.126 (2)	0.0116 (10)	0.0160 (13)	0.0335 (14)
O9	0.0484 (14)	0.0574 (15)	0.122 (2)	0.0277 (12)	0.0034 (14)	0.0368 (15)
O10	0.0321 (12)	0.0536 (14)	0.1036 (19)	0.0167 (10)	0.0065 (12)	0.0268 (13)
N1	0.082 (2)	0.080 (2)	0.054 (2)	0.0174 (18)	−0.0012 (17)	0.0185 (17)
N2	0.086 (2)	0.0566 (19)	0.071 (2)	0.0247 (17)	0.0272 (18)	0.0006 (16)
C1	0.070 (3)	0.056 (2)	0.095 (3)	0.0055 (19)	0.008 (2)	0.031 (2)
C2	0.053 (2)	0.052 (2)	0.060 (2)	0.0240 (17)	0.0074 (16)	0.0210 (16)
C3	0.039 (2)	0.095 (3)	0.097 (3)	0.024 (2)	0.0019 (19)	0.015 (2)
C4	0.0483 (19)	0.054 (2)	0.0447 (18)	0.0195 (16)	0.0031 (14)	0.0065 (15)
C5	0.0466 (18)	0.0496 (18)	0.0354 (16)	0.0211 (14)	0.0027 (13)	0.0076 (13)
C6	0.0463 (18)	0.0498 (18)	0.0391 (17)	0.0234 (14)	0.0016 (13)	0.0140 (14)
C7	0.0435 (17)	0.0455 (17)	0.0384 (16)	0.0203 (14)	0.0013 (13)	0.0121 (13)
C8	0.0399 (16)	0.0476 (18)	0.0424 (17)	0.0208 (14)	0.0026 (13)	0.0104 (14)
C9	0.0484 (18)	0.0467 (18)	0.0356 (16)	0.0282 (14)	0.0029 (13)	0.0100 (13)
C10	0.0507 (19)	0.0427 (17)	0.0421 (17)	0.0190 (14)	0.0020 (14)	0.0087 (13)
C11	0.0367 (15)	0.0290 (14)	0.0402 (16)	0.0120 (12)	0.0027 (12)	0.0109 (12)
C12	0.0387 (16)	0.0300 (15)	0.0505 (18)	0.0163 (12)	0.0004 (13)	0.0109 (13)
C13	0.0371 (15)	0.0272 (14)	0.0470 (18)	0.0087 (12)	0.0040 (13)	0.0074 (12)
C14	0.0389 (16)	0.0318 (15)	0.0446 (17)	0.0054 (12)	0.0005 (13)	0.0135 (13)
C15	0.0356 (15)	0.0354 (15)	0.0513 (18)	0.0135 (13)	0.0029 (13)	0.0195 (13)
C16	0.0283 (14)	0.0265 (14)	0.0493 (18)	0.0101 (11)	0.0082 (12)	0.0127 (12)
C17	0.0505 (19)	0.0365 (17)	0.0507 (19)	0.0117 (14)	0.0047 (15)	0.0058 (14)
C18	0.0485 (19)	0.0425 (18)	0.052 (2)	0.0108 (14)	0.0024 (15)	0.0151 (15)
C19	0.0315 (19)	0.082 (3)	0.135 (4)	0.0209 (18)	0.005 (2)	0.037 (3)
C20	0.0372 (17)	0.0388 (17)	0.065 (2)	0.0164 (14)	0.0021 (15)	0.0103 (15)
C21	0.042 (2)	0.054 (2)	0.133 (4)	0.0035 (17)	0.024 (2)	0.024 (2)
C22	0.0426 (17)	0.0368 (17)	0.058 (2)	0.0137 (14)	0.0111 (14)	0.0116 (14)
C23	0.0390 (16)	0.0352 (15)	0.0458 (17)	0.0151 (13)	0.0076 (13)	0.0129 (13)
C24	0.0336 (16)	0.0441 (17)	0.0582 (19)	0.0209 (13)	0.0106 (14)	0.0159 (14)
C25	0.0403 (16)	0.0339 (15)	0.0460 (17)	0.0209 (13)	0.0110 (13)	0.0159 (13)
C26	0.0354 (15)	0.0314 (15)	0.0501 (18)	0.0126 (12)	0.0073 (13)	0.0114 (13)
C27	0.0347 (15)	0.0357 (15)	0.0444 (17)	0.0163 (12)	0.0035 (12)	0.0089 (13)
C28	0.0411 (17)	0.0316 (15)	0.0508 (18)	0.0196 (13)	0.0047 (13)	0.0126 (13)
C29	0.061 (2)	0.068 (2)	0.087 (3)	0.028 (2)	0.011 (2)	0.018 (2)

### Geometric parameters (Å, °)

**Table:**

Cl1—C29	1.743 (4)	C7—C8	1.383 (4)
Cl2—C29	1.744 (4)	C8—C9	1.381 (4)
Cl3—C29	1.747 (4)	C8—H8	0.9300
O1—C16	1.369 (3)	C9—C10	1.372 (4)
O1—C25	1.403 (3)	C10—H10	0.9300
O2—C11	1.362 (3)	C11—C12	1.383 (4)
O2—C9	1.411 (3)	C11—C16	1.392 (4)
O3—C2	1.191 (4)	C12—C13	1.394 (4)
O4—C2	1.320 (4)	C12—H12	0.9300
O4—C1	1.449 (4)	C13—C14	1.400 (4)
O5—C4	1.203 (4)	C13—C17	1.438 (4)
O6—C4	1.322 (4)	C14—C15	1.389 (4)
O6—C3	1.452 (4)	C14—C18	1.440 (4)
O7—C22	1.196 (4)	C15—C16	1.385 (4)
O8—C22	1.327 (4)	C15—H15	0.9300
O8—C21	1.450 (4)	C19—H19A	0.9600
O9—C20	1.199 (4)	C19—H19B	0.9600
O10—C20	1.320 (4)	C19—H19C	0.9600
O10—C19	1.442 (4)	C20—C27	1.488 (4)
N1—C18	1.135 (4)	C21—H21A	0.9600
N2—C17	1.139 (4)	C21—H21B	0.9600
C1—H1A	0.9600	C21—H21C	0.9600
C1—H1B	0.9600	C22—C23	1.486 (4)
C1—H1C	0.9600	C23—C28	1.382 (4)
C2—C7	1.493 (4)	C23—C24	1.395 (4)
C3—H3A	0.9600	C24—C25	1.377 (4)
C3—H3B	0.9600	C24—H24	0.9300
C3—H3C	0.9600	C25—C26	1.373 (4)
C4—C5	1.492 (4)	C26—C27	1.396 (4)
C5—C6	1.383 (4)	C26—H26	0.9300
C5—C10	1.395 (4)	C27—C28	1.379 (4)
C6—C7	1.395 (4)	C28—H28	0.9300
C6—H6	0.9300	C29—H29	0.9800
			
C16—O1—C25	116.6 (2)	C15—C14—C18	119.7 (3)
C11—O2—C9	117.2 (2)	C13—C14—C18	120.3 (3)
C2—O4—C1	116.7 (3)	C16—C15—C14	120.0 (3)
C4—O6—C3	116.7 (3)	C16—C15—H15	120.0
C22—O8—C21	116.1 (3)	C14—C15—H15	120.0
C20—O10—C19	116.2 (3)	O1—C16—C15	122.6 (2)
O4—C1—H1A	109.5	O1—C16—C11	117.3 (2)
O4—C1—H1B	109.5	C15—C16—C11	120.0 (3)
H1A—C1—H1B	109.5	N2—C17—C13	178.1 (4)
O4—C1—H1C	109.5	N1—C18—C14	178.9 (4)
H1A—C1—H1C	109.5	O10—C19—H19A	109.5
H1B—C1—H1C	109.5	O10—C19—H19B	109.5
O3—C2—O4	123.5 (3)	H19A—C19—H19B	109.5
O3—C2—C7	124.3 (3)	O10—C19—H19C	109.5
O4—C2—C7	112.3 (3)	H19A—C19—H19C	109.5
O6—C3—H3A	109.5	H19B—C19—H19C	109.5
O6—C3—H3B	109.5	O9—C20—O10	123.5 (3)
H3A—C3—H3B	109.5	O9—C20—C27	123.7 (3)
O6—C3—H3C	109.5	O10—C20—C27	112.8 (2)
H3A—C3—H3C	109.5	O8—C21—H21A	109.5
H3B—C3—H3C	109.5	O8—C21—H21B	109.5
O5—C4—O6	123.8 (3)	H21A—C21—H21B	109.5
O5—C4—C5	124.0 (3)	O8—C21—H21C	109.5
O6—C4—C5	112.2 (3)	H21A—C21—H21C	109.5
C6—C5—C10	119.9 (3)	H21B—C21—H21C	109.5
C6—C5—C4	122.6 (3)	O7—C22—O8	123.6 (3)
C10—C5—C4	117.4 (3)	O7—C22—C23	123.9 (3)
C5—C6—C7	120.1 (3)	O8—C22—C23	112.4 (2)
C5—C6—H6	119.9	C28—C23—C24	119.0 (3)
C7—C6—H6	119.9	C28—C23—C22	118.5 (2)
C8—C7—C6	120.3 (3)	C24—C23—C22	122.6 (3)
C8—C7—C2	121.6 (3)	C25—C24—C23	119.5 (3)
C6—C7—C2	118.0 (3)	C25—C24—H24	120.2
C9—C8—C7	118.3 (3)	C23—C24—H24	120.2
C9—C8—H8	120.8	C26—C25—C24	122.1 (2)
C7—C8—H8	120.8	C26—C25—O1	118.6 (2)
C10—C9—C8	122.7 (3)	C24—C25—O1	119.2 (2)
C10—C9—O2	118.9 (3)	C25—C26—C27	118.2 (3)
C8—C9—O2	118.4 (3)	C25—C26—H26	120.9
C9—C10—C5	118.6 (3)	C27—C26—H26	120.9
C9—C10—H10	120.7	C28—C27—C26	120.5 (3)
C5—C10—H10	120.7	C28—C27—C20	117.0 (2)
O2—C11—C12	123.7 (2)	C26—C27—C20	122.5 (3)
O2—C11—C16	116.0 (2)	C27—C28—C23	120.8 (2)
C12—C11—C16	120.3 (3)	C27—C28—H28	119.6
C11—C12—C13	119.9 (2)	C23—C28—H28	119.6
C11—C12—H12	120.0	Cl1—C29—Cl2	109.5 (2)
C13—C12—H12	120.0	Cl1—C29—Cl3	109.9 (2)
C12—C13—C14	119.7 (3)	Cl2—C29—Cl3	110.2 (2)
C12—C13—C17	119.1 (3)	Cl1—C29—H29	109.1
C14—C13—C17	121.2 (3)	Cl2—C29—H29	109.1
C15—C14—C13	120.0 (3)	Cl3—C29—H29	109.1

### Hydrogen-bond geometry (Å, °)

**Table:**

Cg2 is the centroid of the C11–C16 ring.

**Table:**

*D*—H···*A*	*D*—H	H···*A*	*D*···*A*	*D*—H···*A*
C15—H15···O7^i^	0.93	2.60	3.455 (4)	154
C29—H29···O3^ii^	0.98	2.26	3.182 (5)	157
C19—H19A···Cg2^iii^	0.98	2.90	3.709 (4)	143

Symmetry codes: (i) −*x*, −*y*+1, −*z*; (ii) −*x*+1, −*y*, −*z*+1; (iii) *x*−1, *y*, *z*.
